# Dimerization of human uridine diphosphate glucuronosyltransferase allozymes 1A1 and 1A9 alters their quercetin glucuronidation activities

**DOI:** 10.1038/srep23763

**Published:** 2016-03-30

**Authors:** Yan-Qing Liu, Ling-Min Yuan, Zhang-Zhao Gao, Yong-Sheng Xiao, Hong-Ying Sun, Lu-Shan Yu, Su Zeng

**Affiliations:** 1 Institute of Drug Metabolism and Pharmaceutical Analysis, Zhejiang Province Key Laboratory of Anti-Cancer Drug Research, College of Pharmaceutical Sciences, Zhejiang University, Hangzhou 310058, China

## Abstract

Uridine diphosphate glucuronosyltransferase 1A (UGT1A) is a major phase II drug-metabolism enzyme superfamily involved in the glucuronidation of endobiotics and xenobiotics in humans. Many polymorphisms in UGT1A genes are reported to inhibit or decrease UGT1A activity. In this study, two UGT1A1 allozymes, UGT1A1 wild-type and a splice mutant, as well as UGT1A9 wild-type and its three UGT1A9 allozymes, UGT1A9*2(C3Y), UGT1A9*3(M33T), and UGT1A9*5(D256N) were single- or double-expressed in a Bac-to-Bac expression system. Dimerization of UGT1A1 or UGT1A9 allozymes was observed via fluorescence resonance energy transfer (FRET) and co-immunoprecipitation analysis. SNPs of UGT1A altered the ability of protein-protein interaction, resulting in differential FRET efficiencies and donor-acceptor *r* distances. Dimerization changed the chemical regioselectivity, substrate-binding affinity, and enzymatic activity of UGT1A1 and UGT1A9 in glucuronidation of quercetin. These findings provide molecular insights into the consequences of homozygous and heterozygous UGT1A1 and UGT1A9 allozymes expression on quercetin glucuronidation.

Uridine diphosphate glucuronosyltransferase (UGT) is a major phase II drug-metabolism enzyme superfamily involved in biotransformation of xenobiotics in humans by catalyzing the transfer of glucuronic acid to hydroxyl, carboxyl, or amine group compounds^1^. UGT1A1 and UGT1A9 are two members from this superfamily and catalyze the metabolism of many endogenous substances and drugs. For example, bilirubin and estradiol, buprenorphine, acetaminophen, quercetin, etoposide^2^, SN-38^3^ are known as substrates of UGT1A1, while 4-hydroxy estrone, acetylenic estrone and retinoic acid, propofol, acetaminophen, retinoic acid, mycophenolic acid^4^, and quercetin are reported as substrates of UGT1A9.

Several polymorphisms in UGT1A genes have been identified to date. Allelic variants caused by single nucleotide polymorphisms (SNPs) or alternatively splices have demonstrated absent or reduced enzymatic activity, leading to variation in the pharmacokinetics or susceptibility to diseases^5^,^6^,^7^,^8^,^9^. A UGT1A1 splice-mutant (UGT1A1_i2) which lacks transferase activity acted as a negative modulator of UGT1A1, decreasing its activity by up to 78%^10^. Three non-synonymous SNPs: UGT1A9^*^2(C3Y), UGT1A9*3(M33T), and UGT1A9*5(D256N), which showed changes in activity on different substrates, were widely investigated^11^,^12^,^13^,^14^. For *S*-flurbiprofen, UGT1A9*2 exhibited higher glucuronidation activity than UGT1A9*1 (wild-type) and UGT1A9*3 showed virtually no activity^11^. UGT1A9*3 resulted in a dramatic decrease in SN-38 glucuronide formation, with 3.8% of wild-type activity. In turn, the glucuronidation of flavopiridol remains unaffected^12^. Moreover, UGT1A9*5 is nonfunctional with regard to SN-38 glucuronidation^13^. UGT1A9*3 exhibited efficient glucuronidation activity against flavopiridol, but exhibited significant decrease in glucuronidation activity against mycophenolic acid^14^. These results indicate substrate-dependent impact on each variant.

UGT1A isoforms were also reported to display differential chemical regioselectivity to substrates. Chen and coauthors have studied the chemical regioselectivity formation of the monoglucuronides of quercetin (7-, 3-, 4′- and 3′-glucuronide) by human recombinant UGT1A9 and UGT1A3. The results indicated that the catalytic efficiency for UGT1A3 was 3′->3->4′->7- and for UGT1A9 was 3->7->3′->4′-glucuronide^15^. The results for glucuronidation of quercetin, luteolin, and kaempferol by wild-type and UGT1A3 variants showed much lower activity for UGT1A3*2, UGT1A3*3, and UGT1A3*5 compared with that of wild-type UGT1A3. However, UGT1A3*4 exhibited an increase in quercetin glucuronidation efficiency and a clear preference to 7- and 3-hydroxyl groups^16^.

Previous studies demonstrated that human UGT1A enzymes may dimerize using two-hybrid screening^17^, co-immunoprecipitation (Co-IP)^18^ or fluorescence resonance energy transfer analysis (FRET)^19^. Double human UGT isoforms stable-expressing cell lines were established and used to determine the interaction between human UGT1A isoforms and other isoforms^20^. Further studies confirmed that human UGT1A1, UGT1A4, and UGT1A6 interact with each other by heterodimerization^21^. Zhang *et al.* determined the frequencies of 28 UGT1A1 genotypes and found that UGT1A1*6/*63 are the most common genotypes in the Tibetan and Han populations. Additionally, the UGT1A1*28 allele is always detected together with UGT1A1*80^22^.

As previous report, enzyme heterozygotes display distinct activities. UGT1A1*6(G211A)/*60(T3279G) and UGT1A1*6(G211A)/*28(A[TA]7TA) are associated with reducing glucuronidation and increasing serum bilirubin in irinotecan-administered Japanese patients with cancer^23^. In the Japanese population 98% of UGT1A9 I399C > T alleles associated with low-activity haplotypes, either UGT1A1*6, *28, or *60, cause a decrease in efficiency of SN-38 metabolism^24^. Considering the differential enzymatic activity among UGT1A heterozygotes, interactions may occur between the wild-type and allelic variants or among allelic variants. However, direct evidence for oligomerization among UGT1A enzymes in wild-type and allelic variants is not available and the effects of diplotype formation needs further investigation.

In this study (as shown in Fig. 1), UGT1A1 allozymes (UGT1A1*1 and one splice mutant UGT1A1*1b) and UGT1A9 allozymes (UGT1A9*1/*2/*3/*5) were over expressed individually or co-expressed with one other variant in insect cell lines using a Bac-to-Bac expression system, respectively. Homodimeric formation among the wild-type enzyme and variants was determined by quantitative FRET and Co-IP. The enzyme activity and differential chemical regioselectivity were detected by glucuronides formation from quercetin to determine whether the protein-protein interactions between UGT1A allozymes altered the enzymatic characteristics.

## Results

### Quantitative FRET analysis

UGT1A-CFP and UGT1A-YFP fusion proteins were expressed in a Bac-to-Bac insect expression system and proteins expression patterns were evaluated by fluorescence microscopy analysis. As shown in Fig. S1, fluorescence of each UGT1A-CFP and UGT1A-YFP fusion protein resembled an endoplasmic reticulum (ER) localization expression pattern. It suggested that fluorescence tagging of UGT1As did not alter the subcellular localization of UGT1A allozymes.

Quantitative FRET analysis has been widely used to study intra- and inter-molecular interactions *in situ*^25^. Sf9 cells were co-infected with UGT1A-CFP and UGT1A-YFP *Baculovirus*, and the cells were evaluated using FRET analysis to determine oligomerization among two forms of UGT1A1 or UGT1A9 *in vivo*. Sf9 cells co-infected with recombinant CFP and YFP *Baculovirus* were used as the negative control, while cells infected with recombinant CFP-linker-YFP *Baculovirus* served as the positive control.

**Figure 2:**
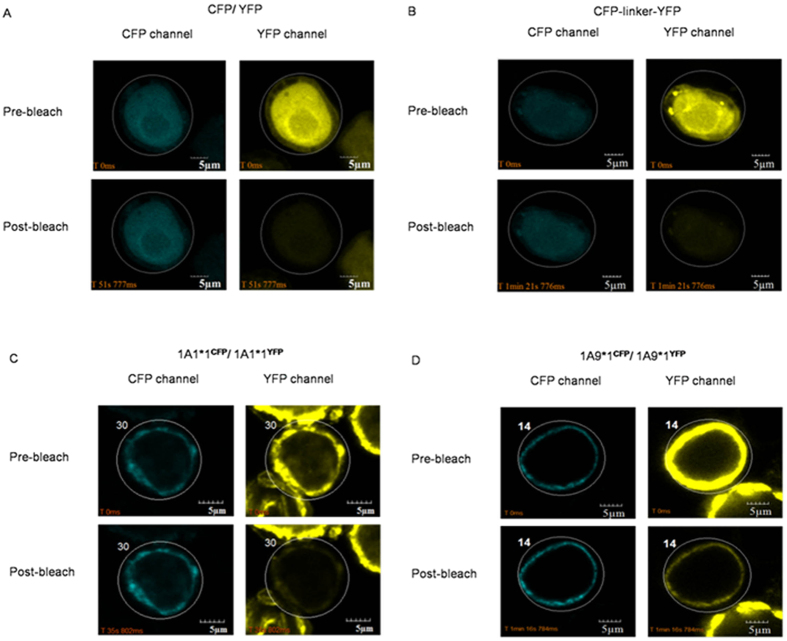
Detection of the FRET phenomenon using the acceptor photobleaching method in UGT1A-CFP and UGT1A-YFP co-infected cells. Sf9 cells were co-infected with recombinant CFP and YFP *Baculovirus* (negative control, **A**) and no increase in CFP fluorescence was observed in the circle zones bleached by the 515 nm laser line. In CFP-linker-YFP (positive control, **B**) UGT1A1*1-CFP + UGT1A1*1-YFP (**C**) and UGT1A1*9-CFP + UGT1A1*9-YFP (**D**)-infected cells, an increase in CFP fluorescence was observed in the circle zones bleached by the 515 nm laser line indicating oligomerization between UGT1A1 or UGT1A9 allozymes.

As shown in Fig. 2, compared to the negative control (Fig. 2A), the fluorescence intensity of CFP channel showed a significant increase in the number of CFP-linker-YFP (Fig. 2B) infected cells and UGT1A1^*^1-CFP/UGT1A1*1-YFP (Fig. 2C) and UGT1A9*1-CFP/UGT1A9*1-YFP (Fig. 2D) co-infected cells. The significant FRET phenomenon indicated that two proteins reside in close proximity to each other within the ER membrane; confirming oligomerization among UGT1A1 or UGT1A9 allozymes. FRET efficiencies and donor-acceptor *r* distances were calculated to visually exhibit the interactions of the two protein isoforms (Table 1). FRET is a powerful technique that can be applied to measure distances between 1 and 10 nm, which is small enough to characterize the proximity of interacting molecules^26^,^27^,^28^. The donor-acceptor *r* distance correlated to each pair of UGT1A1 or UGT1A9 allozymes revealed a dimeric distance^29^. These results suggested that, all UGT1A1 and UGT1A9 allozymes used in this study were capable of oligomerization. It has been reported that FRET donor-acceptor distance is positively associated with tagged domain-domain distance^30^, in other words, FRET donor-acceptor distance reflects the interaction ability between target proteins. Interestingly, the donor-acceptor distance of two non-functional UGT1A1*1b was slightly smaller than wild-type UGT1A1; conversely, UGT1A1*1/UGT1A1*1b dimer showed weaker interaction than UGT1A1*1/1A1*1 dimer. This suggests that the alternative splice is associated with interaction ability of UGT1A1. In UGT1A9 FRET analysis, UGT1A9*1/1A9*1 exhibited shorter donor-acceptor *r* distance and stronger interaction than the other three self-interaction dimers. UGT1A9*1/1A9*3 and UGT1A9*1/1A9*5 showed stronger interactions than that of UGT1A9*3/1A9*3 and UGT1A9*5/1A9*5. This indicates that the interaction ability is somewhat decreased among mutants and the loss of interaction can be partially recovered by interacting with wild-type protein. UGT1A9*2/1A9*3 and UGT1A9*2/1A9*5 exhibited similar interaction ability to UGT1A9*3/1A9*3 and UGT1A9*5/1A9*5. Moreover, the interaction between UGT1A9*1 and UGT1A9*2 was weakened. However, the interaction between UGT1A9*3 and UGT1A9*5 was stronger than that of UGT1A9*5/1A9*5. It seemed that all of the mutants in UGT1A1 and UGT1A9 showed different spatial structure with wild-type protein, and changed the interaction ability of UGTs. The diversity of spatial structure might be associated with substrate specificity and enzymatic activity.

### Co-immunoprecipitation analysis

**Figure 3:**
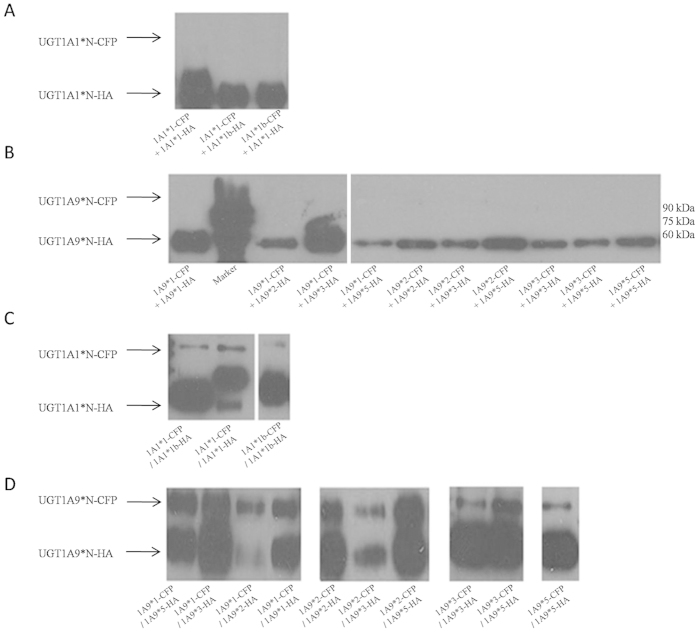
Analysis of the dimerization of UGT1As by Co-IP. The HA-tagged and CFP-tagged proteins were directly mixed and no CFP-tagged protein bands were observed after Co-IP (**A**,**B**). The HA- and CFP-tagged proteins were co-expressed in host cells followed by anti-HA beads immunoprecipitation and western blotting detection, wherein two bands (56 kDa and 81 kDa) were observed (**C**,**D**).

The HA- and CFP-tagged fusion proteins were expressed in Sf9 cells individually. Solubilized proteins were mixed with anti-HA beads, and resuspended protein was detected by western blotting using anti-UGT1A antibody (Fig. S2). As a result, only HA-tagged proteins (56 kDa) were pulled down and revealed bands in western blotting, suggesting that CFP-tagged (81 kDa) proteins were not immunoprecipitated with beads. The HA-tagged and CFP-tagged proteins were directly mixed, and no CFP-tagged protein bands were observed after Co-IP as well (Fig. 3A,B). Furthermore, the HA- and CFP-tagged proteins were co-expressed in host cells followed by anti-HA beads immunoprecipitation and western blotting detection. Interestingly, two bands (56 kDa and 81 kDa) were observed, which suggested that the HA-tagged protein immunoprecipitated with CFP-tagged protein (Fig. 3C,D). As no signals were detected in directly mixed samples, intracellular environment was therefore necessary for the interaction between two forms of UGT1A. Co-IP analysis strengthened the results of FRET analysis and supported direct evidence for dimerization among UGT1A1 or UGT1A9 allozymes.

### Glucuronide formationfrom quercetin

#### Expression levels of UGT1A1 and UGT1A9 allozymes

Double UGT1A1 or UGT1A9 allozymes expressed in Sf9 cells were established to detect changes in enzymatic characteristics. The protein expression levels of UGT1A1 and UGT1A9 allozymes in single- and double-expression systems were evaluated by immunoblot analysis (Fig. S3). The total cell homogenates from Bac-to-Bac expression systems were subjected to 10% sodium dodecyl sulfate-polyacrylamide gel electrophoresis (SDS-PAGE) and the membranes were probed with anti-UGT1A antibody (Fig. S3A−3H). The relative expression levels of UGT1A allozymes were used to normalize the glucuronidation activity.

#### Kinetic analysis of quercetin glucuronide formation in single-expression models

**Figure 4:**
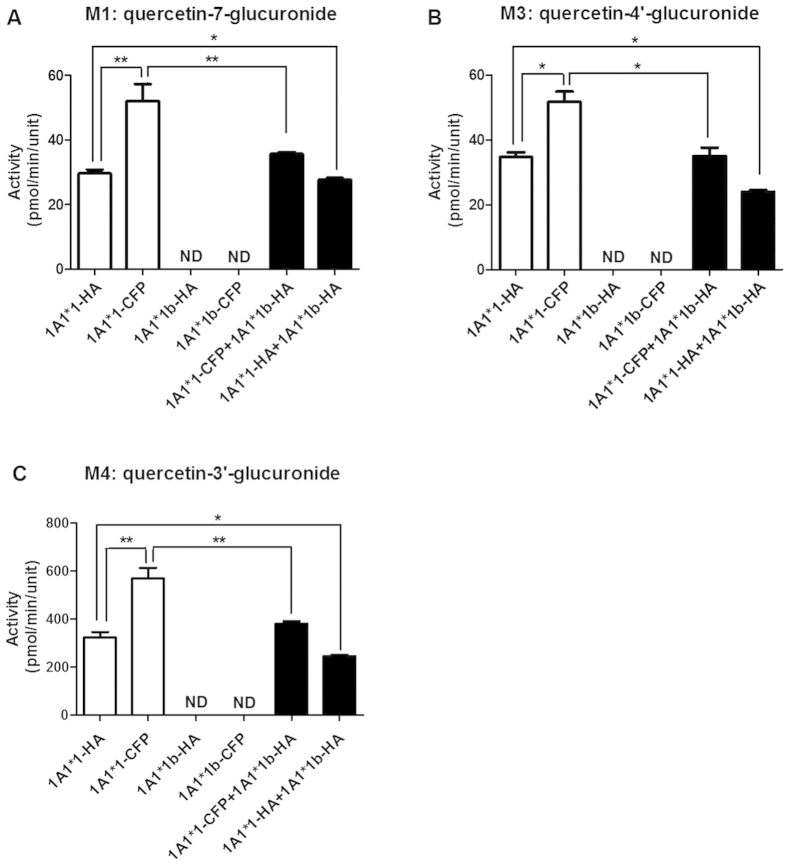
Activity assays of UGT1A1*N or UGT1A1*N-UGT1A1*N on the glucuronidation of quercetin. The concentration of quercetin in the incubation mixture was 100 μmol/L. ☐ indicates single expression and ▄ indicates double expression. Data are presented as mean ± SD from three independent determinations, and the asterisks indicate differences that are statistically significant (**P < 0.005, *P < 0.05).

The hydrolyzed glucuronides were quantified using a modified high performance liquid chromatography (HPLC) method. The quercetin glucuronidation activity was measured in terms of the peak area of targeted glucuronide conjugated compounds. Quercetin was incubated with UGT1A1*1/*1b and UGT1A9*1/*2/*3/*5 from single-expression system and kinetic parameters were estimated. The glucuronidation positions of quercetin monoglucuronides were accorded to our previous study^15^. As a result, quercetin was converted into three main monoglucuronides (M1: 7-glucuronide, M3: 4′-glucuronide, and M4: 3′-glucuronide) by UGT1A1 (Fig. S4A), and the splice mutant UGT1A1*1b showed no enzymatic activity (Fig. 4).

Four monoglucuronides were detected in the UGT1A9 incubation mixture and the M1 (7-glucuronide), M2 (3-glucuronide), and M4 (3′-glucuronide) were the main metabolites (Fig. S4B). We determined the enzyme kinetic parameters of M1 and M2 formation catalyzed by HA- and CFP-tagged UGT1A9 allozymes in single expression systems. As shown in Tables 2 and S1, except for M2 formation catalyzed by HA-UGT1A9*2, UGT1A9*2/*3/*5 displayed significantly decreased M1, M2, and M4 formation compared to the wild-type UGT1A9. Glucuronidation activity was found to be 100%, 24.71%, 21.96%, and 6.65% for M1 formation for HA-tagged UGT1A9*1, 1A9*2, 1A9*3, and 1A9*5 and it was 100%, 66.82%, 24.69%, and 5.56% for M2 formation, respectively. Compared with HA-tagged proteins, the glucuronidation activity of CFP-tagged UGT1A9*1, 1A9*2, 1A9*3 and 1A9*5 was 130.5%, 20.48%, 15.12%, and 22.08% toward M1 formation and 264.1%, 9.64%, 23.10%, and 14.95% for M2 formation, respectively. Moreover, the HA/CFP-tagged UGT1A9*2 and 1A9*3 also exhibited differential affinity with wild-type UGT1A9: both of them showed weaker affinity in M1 formation, but 1A9*2-HA displayed significant higher affinity in M2 formation. Both HA- and CFP-tagged UGT1A9*2, 1A9*3, and 1A9*5 proteins demonstrated considerable decrease in transferase ability for M4 formation than UGT1A9*1 (Fig. S5).

**Figure 5:**
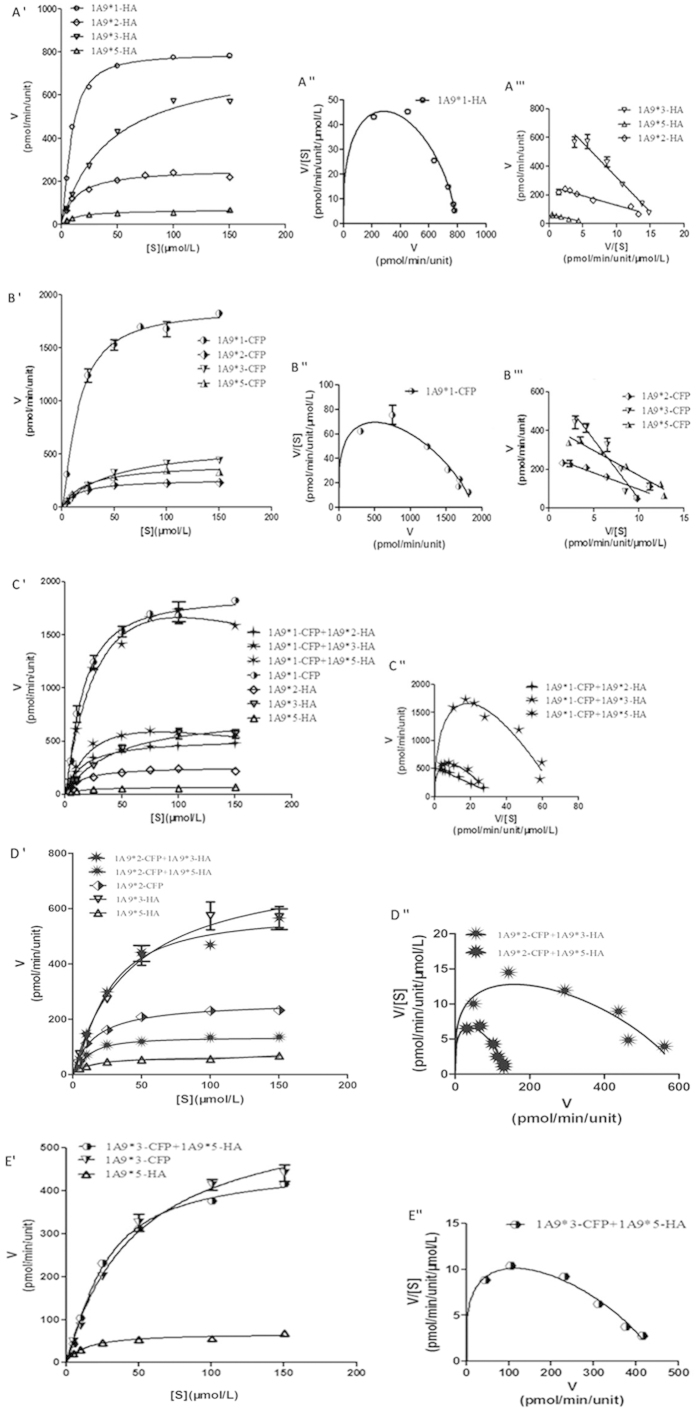
Enzyme kinetic, Eadie-Hofstee, and *V*/*S*−*V* plots for quercetin-7-glucuronidation by UGT1A9*N-HA, UGT1A9*N-CFP, and UGT1A9*N-CFP + UGT1A9*N-HA. The glucuronidation rates are presented as mean ± SD of three independent determinations. The rates were normalized according to the relative expression levels. The respective kinetic constants are presented in Tables 2 and 3.

**Figure 6:**
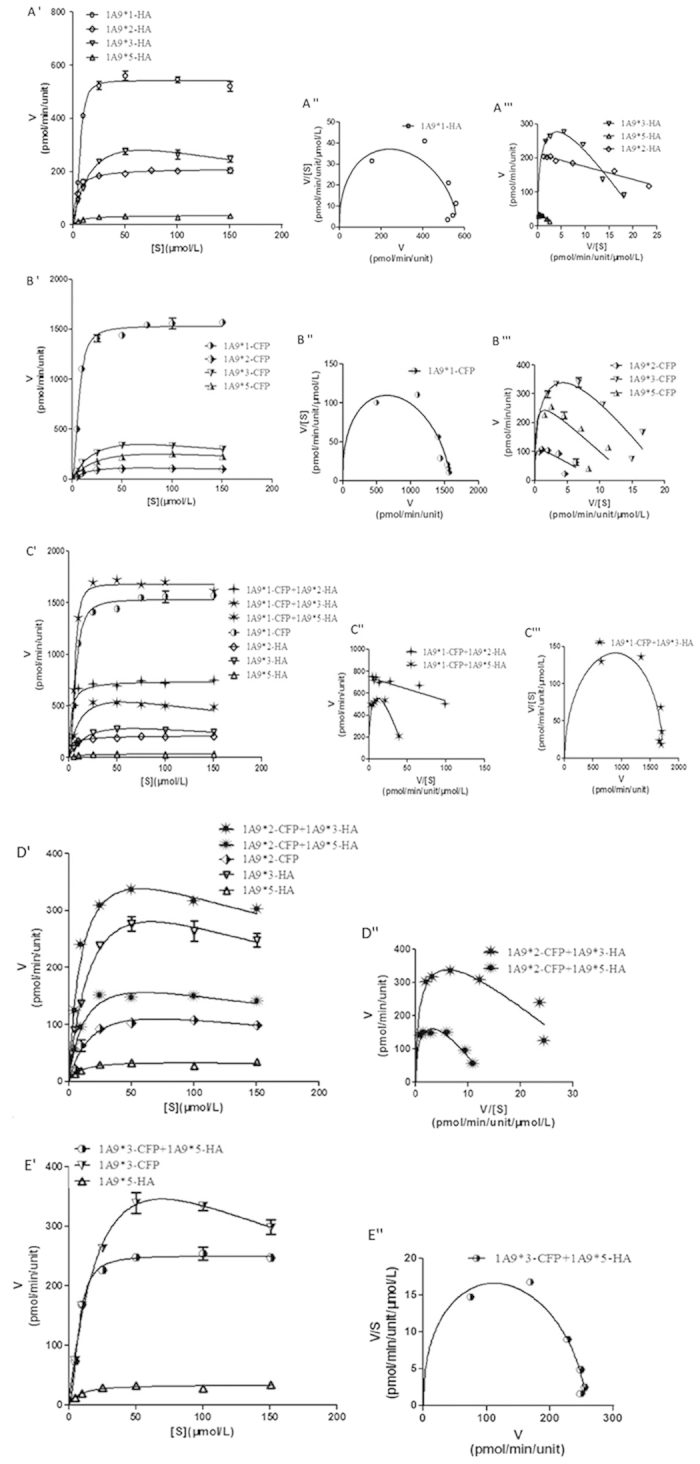
Enzyme kinetic, Eadie-Hofstee, and *V*/*S*−*V* plots for quercetin-3-glucuronidation by UGT1A9*N-HA, UGT1A9*N-CFP, and UGT1A9*N-CFP + UGT1A9*N-HA. The glucuronidation rates are presented as mean ± SD of three independent determinations. The rates were normalized according to the relative expression levels. The respective kinetic constants are presented in Tables S1 and S2.

In the context of HA-tagged constructs (Figs 5 and 6), the kinetic parameters of UGT1A9*1 were found to exhibit allosteric effects on M1 and M2 formation. However, the kinetic parameters were found to fit well with Michaelis-Menten kinetics of UGT1A9*2, 1A9*3, and 1A9*5 for M1 formation and UGT1A9*2 and 1A9*5 for M2 formation. In the context of the CFP-tagged constructs (Figs 5 and 6), the kinetic parameters of UGT1A9*2, 1A9*3, and 1A9*5 were found to fit well with Michaelis-Menten kinetics for M1 formation. Interestingly, CFP-tagged UGT1A9*2 and 1A9*3 displayed similar kinetic parameters as UGT1A9*1 for M2 formation.

These results suggest that the SNPs altered substrate binding ability and chemical regioselectivity of UGT1A9. The fused HA or CFP tag also influenced the kinetic parameters of UGT1A9 to some extent.

#### Kinetic analysis of quercetin glucuronide formation in double-expression models

Alteration of enzyme characteristics by protein-protein interactions of UGT1A allozymes was evaluated using double-expression models. UGT1A1 exhibited a significant loss of enzymatic activity when co-expressed with UGT1A1*1b, which lacks transferase ability (Fig. 4). This implies that UGT1A1*1b decreased the activity of UGT1A1 via protein-protein interaction. UGT1A1*1b displayed a similar role in with another splice mutant, where it reportedly acted as a negative modulator of UGT1A1^10^.

The kinetic parameters of each pair of UGT1A9 allozymes were estimated (Figs 5 and 6, Tables 3 and S2). The activity of UGT1A9*2-HA in M1 and M2 formation significantly increased when co-expressed with UGT1A9*1-CFP, but the kinetic parameters were found to fit Michaelis-Menten kinetics, which was not similar to wild-type UGT1A9. Co-expression of UGT1A9*1-CFP and UGT1A9*3-HA significantly increased M1 and M2 formation and the kinetic parameters were similar to that of wild-type. The activity of UGT1A9*1-CFP/*5-HA showed no changes for M1 formation, but increased for M2 formation. UGT1A9*2-CFP/*3-HA and UGT1A9*2-CFP/*5-HA displayed a significant decrease of *CL*_int_ values for M1 formation, showing kinetic parameters similar to UGT1A9*1.

The UGT1A9*2-CFP/*3-HA and UGT1A9*2-CFP/*5-HA enhanced the activity of M1 and M2 formation. The interaction of UGT1A9*3-CFP and UGT1A9*5-HA occurred functional complementation, and increased the *CL*_int_ values in both M1 and M2 formation, and UGT1A9*3/1A9*5 exhibited similar characteristics with UGT1A9*3. Similar phenomena were observed in UGT1A9*1/1A9*3 and UGT1A9*2/1A9*3 co-expression models, suggesting that the interaction among UGT1A9*1/1A9*3, UGT1A9*2/1A9*3, UGT1A9*2/1A9*5, and UGT1A9*3/1A9*5 altered their three-dimensional structures relative to each other and resulted in wild-type-like kinetic parameters. As shown in Fig. S5B, except for UGT1A9*1-CFP/*5-HA, the other interactions of UGT1A9 allozymes resulted in higher activity than the corresponding theoretical values for M4 formation. This confirms that interaction occurs between any two forms of UGT1A9 allozyme.

In single-expression models (Table 2 and S1), UGT1A9 allozymes exhibited different affinity to quercetin during M1 and M2 formation. In double-expression models (Table 3 and S2), most of the UGT allozyme pairs exhibited lower affinity than the high-affinity allozyme in each group; however, UGT1A9*1-CFP/*2-HA, UGT1A9*1-CFP/*3-HA, and UGT1A9*2-CFP/*3-HA displayed higher affinity than the high-affinity allozyme during M2 and M4 formation. Interestingly, both interactions displayed a preference to 3-glucuronide.

The interaction between two forms of UGT1A9s might change the three-dimensional structure of each protein to alter the substrate-binding domain and the subsequent enzymatic activity. However, interactions between different UGT1A9 allozymes resulted in changeable spatial conformation and differential enzymatic characteristics.

## Discussion

Accumulating evidence to date shows that UGT enzymes exist as oligomers in homodimeric and heterodimeric states^19^,^20^,^31^,^32^. The homodimerization of UGT1A1, UGT1A9, and other UGT1A subfamily proteins has been determined by using FRET^19^ and Co-IP^18^. Our lab recently reported the dimerization of two UGT2B7 allozymes^33^. However, evidence for oligomerization of wild-type and allelic UGT1A variants is not fully described and the glucuronidation activity changes based on the interaction between two allozymes needs further investigation.

In this study, the formation of hetero-dimers among two forms of UGT1A1 or UGT1A9 allozymes was confirmed by FRET and Co-IP. The different UGT1A1 or UGT1A9 allozyme dimers showed distinct differences in donor-acceptor distances, indicating differences in interaction ability. Furthermore, the altered glucuronidation activities of these dimers on substrate quercetin were estimated. Considering the correlation between donor-acceptor distances *r* and enzyme kinetics, we conclude that each mutant detected in this study changed the interaction ability of UGT1A allozymes and altered enzymatic activity via conformational change. A similar phenomenon was also previously observed in the oligomerization among two UGT isoforms^21^.

It is well-known that appropriate three-dimensional structure is necessary for substrate binding and protein-protein interaction. Mutations of amino acid residues always result in changes in the crystal structure of the enzymes and alteration of enzymatic activity. An SNP at codon 183 (Cys > Gly) was identified to have changed glucuronidation activity compared with wild-type UGT1A9 and this mutant variant was then incapable of homodimerization^14^. Compared with UGT1A1, deficiencies of the enV2 helix and the transmembrane helix were found in the UGT1A1*1b sequence (Fig. S6). UGT1A1*1b mutation disrupts the hydrophobic packing of the protein^34^, resulting in loss of activity. The variable N-terminal sequence is generally considered to play a major role in substrate binding, while the highly conserved C-terminal domain probably harbors the UDP-glucuronic acid (UDPGA) binding site. Both UGT1A9 mutations we tested in this study are located in the N-terminal domain. As previously reported, substitution of Met33 variably affected kinetics and catalytic efficiency of UGT1A9. Compared to wild-type UGT1A9, the *K*_m_ values were generally higher, whereas *V*_max_ and *CL*_int_ values were generally lower when incubated with different substrates, indicating a pivotal role for residue-33 in substrate binding^35^. Substituting M33 with T resulted in a significant increase in the value of *K*_m_ for glucuronide formation (Tables 2, 3, S1 and S2). In the UGT1A1 molecular model, the corresponding site of M33(V35 in UGT1A1) is close to the substrate-binding domains Nα3 and Nα5^35^. D256, located between Nβ6 and Nβ7, has an interaction with enV2, and might be associated with hydrophobic packing. In a single expression system, UGT1A9*2-HA (CFP) showed lower activity in quercetin-7-glucuronide formation than the wild-type protein. However, during quercetin-3-glucuronide formation, the HA-tagged variant exhibited higher transfer activity. On the contrary, the CFP-tagged protein showed lower activity, but exhibited substrate inhibition ability. It is challenging to determine the mechanism of how different tags alter the function of UGT1A9*2.

In co-immunoprecipitation analyses, UGT1A allozymes formed dimers upon co-expression in Sf9 cells but not in direct mix of lysates. Similar phenomenon was previously observed^19^,^33^. This suggests that coordinated synthesis in the cell is necessary for the dimerization of UGTs. We determined FRET efficiencies of UGT1A9*1-CFP/*1-YFP in 1% methanol-, quercetin-, and UDPGA-treated cells. Interestingly, compared to 1% methanol and UDPGA treatment, quercetin weakened the interaction ability of UGT1A1 with lower FRET efficiencies and larger donor-acceptor distances (Fig. 2) and diffused protein was also observed. This proved that binding of quercetin changed the characteristics of UGT1A9 and was involved in protein-protein interaction. This phenomenon can be explained by allosteric effects in enzymes. UGT1A9*2 (C3Y) exhibited a higher *V*_max_, *K*_m_, and intrinsic clearance toward (S)-Flurbiprofen than UGT1A9*1^11^ but exhibited similar activity as the wild-type toward both, SN-38 and flavopiridol^36^. It seems that the mutant changed allosteric effects in UGT1A9 and as a result, UGT1A allozymes showed differential substrate specificity.

As previously reported, UGT1A variants displayed differential enzymatic activity and chemical regioselectivity to substrates. UGT1A1*1b showed no activity of quercetin glucuronide formation; however, UGT1A9 variants exhibited diverse catalytic efficiency of quercetin glucuronidation. Interestingly, the mutation and dimerization were both involved in the change of regioselectivity of UGT1A9, where UGT1A9*1 exhibited preference to 7-glucuronide and UGT1A9*2 exhibited preference to 3-glucuronide, UGT1A9*3 and UGT1A9*5 showed similar preference to 7-glucuronide and 3-glucuronide, and the combination of two allozymes displayed a preference to 3-glucuronide. Compared with UGT1A1*1/UGT1A1*1 single expression allozyme, UGT1A1*1/UGT1A1*1b combination exhibited significantly lower activity toward quercetin because of the negative modulation of UGT1A1*1b on UGT1A1*1. For 7-glucuronide and 3-glucuronide formation, the main conjugate products by UGT1A9* toward quercetin, UGT1A9*1-CFP/*2-HA and UGT1A9*1-CFP/*3-HA combinations both showed higher activity than theoretical algebra sum of single expression allozymes, suggesting the cooperative protein-protein interaction. Also for 7-glucuronide and 3-glucuronide formation, UGT1A9*3-CFP/*5-HA combination displayed gain of function compared with each other, indicating the interaction between them and the formation of a functional complex. The mechanism of kinetic parameter alterations via SNP and protein-protein interactions are complex and further studies are necessary. In addition, according to the results of the present *in vitro* study, we believe that the homozygous and heterozygous dimerization of UGT1A enzymes may exist *in vivo* which could affect the UGT1A activity. Unfortunately, the dimerization *in vivo* was hard to detect now. Therefore, it is an interesting but difficult challenge to study the effect of dimerization to the activity of UGT1A *in vivo*.

In conclusion, our study demonstrated the oligomerization of UGT1A1 and UGT1A9 allozymes by FRET and Co-IP. The interaction was complex, depending on isoforms and substrates. Mutations in UGT1A and quercetin-binding altered the affinity of protein-protein interaction, resulting in differential FRET efficiencies. These mutants also showed differential chemical regioselectivity and multiple enzymatic characteristics. We deliberated that the mutants exhibited different characteristics from wild-type UGTs via altered three-dimensional structure of the protein. Additional amino acid substitutions should be performed to elucidate the molecular mechanism of UGTs and increase our understanding of this important enzyme family.

## Materials and Methods

### Materials

Quercetin (chemical purity >98.5%) was purchased from National Institute for Food and Drug Control (Beijing, China). UDPGA, alamethicin and β-D-glucuronidase were purchased from Sigma Chemical Co. (St. Louis, MO, USA). The pGEM-T plasmid was purchased from Promega (Madison, WI, USA). The pECFP-N1 plasmid and pEYFP-N1 plasmid were purchased from Clontech Laboratories, Inc (Palo Alto, CA, USA). Restriction endonucleases, DNA molecular marker, PrimeScript RT reagent kit and T4 ligase were obtained from TaKaRa Bio Inc. (Dalian, Liaoning, China). Cellfectin II reagent, pFastBac1 vector, *E. coli* DH10Bac cells, Sf900II SFM, and Gibco fetal bovine serum were purchased from Invitrogen Corp. (Carlsbad, CA, USA). Spodoptera frugiperda Sf9 insect cells were obtained from the China Center for Type Culture Collection (Wuhan, China). Rabbit Anti-UGT1A Polyclonal antibody was obtained from Institute of Genetics and Developmental Biology, Chinese Academy of Sciences (Beijing, China). The anti-HA beads were purchased from Roche Applied Science (Indianapolis, IN, USA). SuperSignal West Pico was obtained from Pierce Chem Co. (Rockford, IL, USA) and *X*-ray film obtained from Kodak (Rochester, NY, USA).

### Construction of the CFP- , YFP- and HA-tagged UGT1A expression plasmids

A pair of PCR primers was designed according to the published UGT1A1 sequence (GenBank accession no. NM_000463) to amplify the targeted UGT1A1 coding sequence from human liver cDNA: forward prime (5′-cgcggatccatggctgtggagtcccagggcgga-3′) containing a *BamH* I site (underlined), and reverse primer (5′-cccaagctttcacatctgtcttcctgactgctgcttct-3′) containing a *Hind* III site (underlined). Total RNA from cells was isolated using TRIzol, and then, the reverse transcriptase enzyme mix was added to produce the cDNA according to the manufacturer’s instructions (PrimeScript RT reagent kit with gDNA Eraser, TaKaRa). The obtained DNA was cloned into the pMD-18T vector. However, the sequencing results suggested that an extra 134 bp fragment (agaaagaagcagcagtcaggaagacagatgtgaagagctggagcatgttcagatgagaggagacggaacacggggacacaccagcttgagcaagggacaacaggggaggactgatgactgacttcccacctttg), which is located in intron 4 of UGT1A1 locus (I1089-I1122), was inserted between the 1302 bp and the 1303 bp of UGT1A1 open reading frame sequence and altered the following reading frame of UGT1A1 protein. We named this UGT1A1 splice variant plasmid pMD-18T-UGT1A1*1b. The members of UGT1 subfamily contain common sequence from exon 2 (with a *Mun* I site) to exon 5(with an *Asc* I site). To generate the pMD-18T-UGT1A1 plasmid, which contained the UGT1A1-wild type sequence, a fragment from PUC18-UGT1A9*1 plasmid was excised by using *Mun* I and *Asc* I, and replaced the corresponding sequence in pMD-18T-UGT1A1*1b. PUC18-UGT1A9*1/*2/*3/*5 plasmids(UGT1A9 cloned into *BamH* I and *Hind* III sites) had been constructed successfully in our laboratory^11^.

Each constructed plasmid, including the UGT1A9*1/*2/*3/*5 was amplified by PCR with new primers:

UGT1A1 F: 5′-acgcgtcgacatggctgtggagtc-3′, R: 5′-cgcggatcc**tg**ggtcctggatttgtg-3′;

UGT1A1*1b F: 5′-acgcgtcgacatggctgtggagtc-3′, R: 5′-cgcggatcc**at**catctgtcttcctgac-3′;

UGT1A9*1/*3/*5 F: 5′-acgcgtcgacatggcttgcacagggtg-3′, R: 5′-cgcggatcc**tg**ggtcctggatttgtg-3′; UGT1A9*2 F: 5′-acgcgtcgacatggcttacacagggtg-3′, R: 5′-cgcggatcc**tg**ggtcctggatttgtg-3′.

Each forward primer (F) containing a *Sal* I site followed by an initiation ATG codon and reverse primer (R) with a *BamH* I site followed by a mutated stop codon shown in bold. After the cloning of these PCR produces into pECFP-N1and pEYFP-N1vectors, the chimeric UGT1A-CFP and UGT1A-YFP DNAs were excised by digested with *Sal* I and *Not* I, and subcloned into the *Sal* I and *Not* I sites in the pFastBac1 vector, producing the pUGT1A-CFP and pUGT1A-YFP plasmids.

pMD-18T-UGT1A1/1A1b and PUC18-UGT1A9*1/*2/*3/*5 plasmids were used to generate chimeric UGT1A-HA DNAs by PCR with forward primer containing a *Sal* I site (underlined) and reverse primer with a *Not* I site (underlined) and the HA sequence (bold) in each pair of primer:

UGT1A1 F: 5′-acgcgtcgacatggctgtggagtc-3′, R: 5′-cgcgcggccgccta**ggcataatctggcacatcataagggtattccat**atgggtcttggat-3′;

UGT1A1*1b F: 5′-acgcgtcgacatggctgtggagtc-3′, R: 5′-cgcgcggccgccta**ggcataatctggcacatcataagggtattccat**catctgtcttcct-3′;

UGT1A9*1/*3/*5 F: 5′-acgcgtcgacatggcttgcacagggtg-3′, R: 5′-cgcgcggccgccta**ggcataatctggcacatcataagggtattccat**atgggtcttggat-3′;

UGT1A9*2 F: 5′-acgcgtcgacatggcttacacagggtg-3′, R: 5′-cgcgcggccgccta**ggcataatctggcacatcataagggtattccat**catctgtcttcct-3′.

The PCR products were then digested with *Sal* I and *Not* I and cloned into pFastBac1 vector, respectively. In all, a list of UGT1A1 and UGT1A9 plasmids were made with each expressing a fusion protein with either CFP, YFP or HA carboxyl-terminal tags (Table S3).

### Expression of CFP, YFP and HA tagged-UGT1A allozymes in Sf9 cells

A Bac-to-Bac system was used in this study to express the fusion proteins. In brief, the recombinant pFastBac1-UGT1A plasmids were transformed into *Escherichia coli* DH10Bac cells respectively. The transposition of UGT1As into bacmids was confirmed by PCR. The recombinant bacmids were then transfected into the 80% confluent Sf9 cells, hosts for expression, by using Cellfectin II reagent. The supernatant was collected to harvest the first passage *Baculovirus* at 72 h post transfection. For further *Baculovirus* propagation, the primary *Baculovirus* was used to infect the hosts and passaged at least three times. High titre *Baculovirus* stocks were stored at −80 °C until used for protein production.

### Analysis of the dimerization of UGT1A1*1 or UGT1A9*1 with its allelic variants by using FRET

In this study, fluorescence resonance energy transfer (FRET) was used to determine the oligomerization of UGT1A1 or UGT1A9 with its allelic variants, respectively. Sf9 monolayer cells were seeded on 6-well culture plates (at a density of 2 × 10^6^ cells/well), and cells were co-infected with recombinant UGT1A1*N-CFP *Baculovirus* and recombinant UGT1A1/9*N-YFP *Baculovirus*. The hosts which co-infected with CFP- and YFP- tagged UGT1A *Baculovirus* were served as the negative controls, and the CFP-linker-YFP *Baculovirus* infected cells acted as the positive control^33^. At 72 h post infection, the cells were rinsed with PBS and fixed with 4% para-formaldehyde (PFA) for 30 min at room temperature. The cells were washed twice with PBS and then mounted on slides. The fluorescence intensity was detected by an Olympus BX61W1-FV1000 confocal microscope (Olympus, Tokyo, Japan). The FRET efficiency and distance (*r*) between the donor and acceptor was calculated by using acceptor photobleaching method as described previously^33^. Briefly, tranfected Sf9 cells were fixed by in 4% PFA and each slide were taken images in the CFP (donor fluorophore, excitation: 405 nm, emitter: 476 nm), and YFP (acceptor fluorophore, excitation: 515 nm, emitter: 527 nm) channels. Firstly, the laser intensity should be adjusted to avoid the detectable photobleaching in CFP (405 nm) and YFP (515 nm) channels (both less than 5%). Secondly, in order to achieve the best possible dynamic range, the gain of the photomultiplier tube (PMT) was adjusted to eliminate the cross-talk between CFP and YFP channel. Lastly, the chosen Sf9 cells were bleached with the 515 nm laser intensity at 100%. Two images of before-photobleaching and eight images of post-photobleaching were detected in the CFP and YFP channels. FRET efficiency (*E*) was calculated by measuring the changing of donor fluorophore emission before and after photobleaching the acceptor. The following equations were used: E = (Da–Db)/Da, where *D*_a_ and *D*_b_ are the donor fluorescence intensities after and before acceptor photobleaching, respectively. The distance (*r*) between the donor and acceptor can be calculated by using equation E = R_0_^6^/ (R_0_^6^ + r^6^), where *R*_0_ is the Forster distance (52.16 Å, manufacture data).

### Co-immunoprecipitation

The Co-immunoprecipitation was carrid out to further validate the oligomerization among UGT1A1 or UGT1A9 allozymes. Confluent Sf9 cells were infected with UGT1A1*N-CFP, UGT1A1*N-HA and UGT1A1*N-CFP + UGT1A1*N-HA (“N” indicates any of the UGT1A1), respectively. Cells cultivated in 6-well culture plates were harvested at 72 h post infection followed by two washes with PBS and then lysed with 1 mL of lysis buffer (0.05 M Tris-HCl, pH 7.4, 0.15 M NaCl, 0.25% deoxycholic acid, 1% Nonidet P-40, 1 mM EDTA) supplemented with 1% protease mixture inhibitor. The entire supernatant from one well was harvested after sedimention at 13,000 × *g* for 30 min at 4 °C followed by adding 30 μL of anti-HA beads. The mixture was incubated at 4 °C on a rocker platform overnight and the anti-HA beads were collected by centrifugation for 1 min at 1,000 × *g*. Washed thrice with cold lysis buffer, the anti-HA beads were resuspended in 60 μL of SDS-PAGE loading buffer and heated at 95 °C for 10 min. The beads were centrifuged for 1 min at 1,000 × *g* again and the supernatant was collected followed by western blot analysis with anti-UGT1A antibody that recognizes UGT1A1, UGT1A1*1b and UGT1A9 allozymes. Same experimental procedures were taken to test the oligomerization among each two UGT1A9 allozymes.

To determine whether intracellular environment was necessary for the interaction between two forms of UGT1A, the CFP- or HA-tagged UGT1A proteins from single expression system were harvested, respectively. The HA-tagged and CFP-tagged proteins were directly mixed and the mixture were incubated with anti-HA beads followed by same experimental procedures as above.

### Western blot analysis

The supernatant from the co-immunoprecipitation were separated on 10% resolving sodium dodecylsulfate (SDS)-polyacrylamide gels, followed by electrophoretic transfer to PVDF membrane for 90 min. The membranes were blocked with 5% BSA in TBS-T overnight at 4 °C, and then incubated with rabbit anti human UGT1A (1:4000 diluted) antibody at room temperature for 2 h. After three washes, the membranes were incubated with 5000-fold diluted goat anti-rat IgG (H + L) for 1 h. Rinsed with TBS-T three times, membranes were visualized using SuperSignal West Pico, and signals were detected by *X*-ray film.

To determine the expression levels of CFP- and HA-tagged UGT1A allozymes in single and double expression systems, 2–8 μg of the total cell homogenates were were subjected to 12% SDS-PAGE and transferred to a nitrocellulose membrane. The UGT1A proteins detected with anti-UGT1A antibody followed by the analysis of the densities of the bands on the membrane by using Odyssey infrared imaging system (LI-COR Biosciences). The UGT1A1 WT^HA^ from single expression system was set to 1 unit/mg in the calibration curve of the relative expression level. The quantification of relative expression levels of CFP- or HA-tagged tagged UGT1A*N allozymes were calculated by comparing the western blot densities to that of the calibration curve, and used for kinetic experiments.

### Glucuronidation of quercetin

Enzyme activity of UGT1A1 was determined by the glucuronide formation of quercetin glucuronides. Sf9 cells, with expression of UGT1A1*N, were harvested at 72 h post infection. Each typical reaction mixture consisted of a total volume of 100 μL containing Tris-HCl (pH = 7.4, 0.1 M), MgCl_2_ (10 mM), alamethicin (50 μg/mg of protein), total cell homogenates (1 mg/mL), and quercetin (0.1 mM). Incubations were preincubation at 37 °C for 5 min followed by adding the UDPGA (5 mM) to active the glucuronidation reaction. Thirty min later, 300 μL leuteolin-added (internal control) methanol was added to terminate the reaction. The mixture was sedimented at 13,000 × *g* for 10 min, and 25 μL of the supernatant was subjected into HPLC system. To detect the enzyme activity and chemical regioselective abilities of UGT1A9*N, each total cell homogenates (1 mg/mL) were incubated with serial dilutions of quercetin (5.011, 10.022, 25.055, 50.110, 100.220, and 150.330 μM) at 37 °C for 30 min.

High performance liquid chromatography (HPLC) analyses were performed on an Agilent 1200 system, equipped with an Agilent Extend^TM^ C18 (250 mm × 4.6 mm, 5 μm) column. The mobile phase consisted of methanol and 0.02 M phosphoric acid, pH 2.0 (46:54, v/v) with a flow rate of 1 mL/min. Quercetin and its metabolites were detected at a wavelength of 368 nm. The HPLC method was validated according to the US Food and Drug Administration guideline^37^.

### Calculation of enzyme kinetics

The kinetic parameters were estimated using GraphPad Prism, version 5.0 (GraphPad Software Inc., San Diego, CA). The following equations were used: the Michaelis-Menten equation, *v = V*_max_·*S*/(*K*_m_ + *S*), and the substrate inhibition model, *v = V*_max_/(1 + (*K*_m_/*S*) + (*S*/*K*_si_)), where *v* is the velocity of the reaction, *S* is the substrate concentration, *K*_m_ is the Michaelis-Menten constant, *V*_max_ is the maximum velocity, and *K*_si_ is the constant describing the substrate inhibition interaction^38^. The intrinsic clearance (*CL*_int_) was calculated by *V*_max_/*K*_m_.

### Statistical analysis

The values represent the means ± standard deviation (SD) for at least triplicate tests. Statistical comparisons were determined by one-way analysis of variance (ANOVA) followed by a Dunnett’s post-hoc test (SPSS 13.0 software, SPSS Inc., Chicago, IL, USA). A value of *P* < 0.05 was considered statistical significant.

## Additional Information

**How to cite this article**: Liu, Y.-Q. *et al.* Dimerization of human uridine diphosphate glucuronosyltransferase allozymes 1A1 and 1A9 alters their quercetin glucuronidation activities. *Sci. Rep.* **6**, 23763; doi: 10.1038/srep23763 (2016).

## Supplementary Material

Supplementary Information

## Figures and Tables

**Figure 1:**
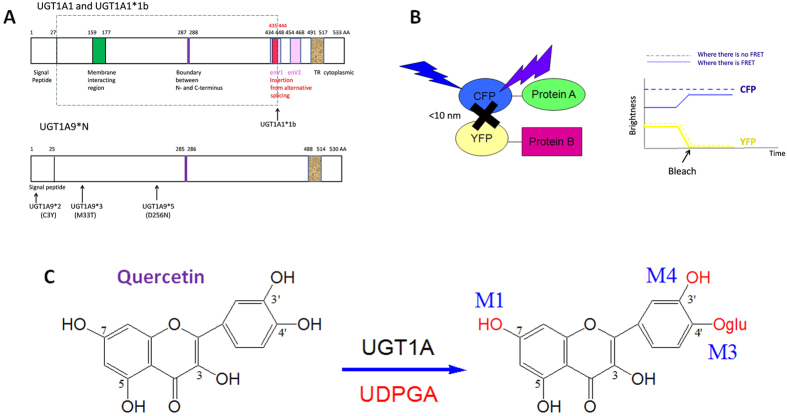
Dimerization of human UGT 1A1 and 1A9 alters their quercetin glucuronidation activities. (**A**) Domains of UGT1A1*1/*1b and UGT1A9*1/*2/*3/*5; (**B**) Dimer detection by FRET using CFP and YFP tags; (**C**) Chemical reaction of quercetin glucuronidation catalyzed by UGT1A.

**Table 1:** Apparent FRET efficiency values *E* and donor-acceptor distances *r* of co-expression proteins.

protein-protein	FRET efficiency *E* %	donor-acceptor distance *r* (nm)	n
CFP/YFP	−1.56 ± 4.8	>10	5
CFP-linker-YFP	17.4 ± 4.8	6.92	21
1A1*1-CFP/1A1*1-YFP	18.3 ± 4.9	6.77	7
1A1*1b-CFP/1A1*1b-YFP	23.6 ± 7.8	6.41	13
1A1*1-CFP/1A1*1b-YFP	14.5 ± 3.4	7.09	19
1A9*1-CFP/1A9*1-YFP	25.1 ± 11.5	6.33	27
1A9*2-CFP/1A9*2-YFP	21.9 ± 3.8	6.52	10
1A9*3-CFP/1A9*3-YFP	13.8 ± 6.5	7.16	8
1A9*5-CFP/1A9*5-YFP	9.6 ± 4.1	7.67	18
1A9*1-CFP/1A9*2-YFP	11.2 ± 4.3	7.45	16
1A9*1-CFP/1A9*3-YFP	19.9 ± 8.6	6.65	20
1A9*1-CFP/1A9*5-YFP	17.0 ± 8.9	6.87	16
1A9*2-CFP/1A9*3-YFP	12.3 ± 4.7	7.32	15
1A9*2-CFP/1A9*5-YFP	9.9 ± 5.8	7.62	12
1A9*3-CFP/1A9*5-YFP	15.5 ± 6.7	7.00	11
1A9*1-CFP/1A9*1-YFP (+1% Methanol)	17.2 ± 8.2	6.86	20
1A9*1-CFP/1A9*1-YFP (+150 μmol/L quercetin)	7.84 ± 3.0	7.96	10
1A9*1-CFP/1A9*1-YFP (+5 mmol/L UDPGA)	22.9 ± 9.9	6.46	13

**Table 2:** Kinetic parameters for quercetin-7-glucuronide by UGT1A9 single expression systems (n = 3).

1A9*N-1A9*N	*K*_m_(μmol/L)	*V*_max_ (pmol/min/unit)	*CL*_int_ (μL/min/unit)	% of 1A9*1-HA	% of 1A9*1-CFP
9*1-HA	10.46 ± 1.20	864.3 ± 23.7	82.6	100	76.62
1A9*2-HA	12.62 ± 1.97	257.6 ± 9.5^***^	20.41^***^	24.71	18.93
1A9*3-HA	42.34 ± 8.23	768.2 ± 66.9	18.14^***^	21.96	16.83
1A9*5-HA	12.40 ± 1.43	68.02 ± 1.98^***^	5.49^***^	6.65	5.09
1A9*1-CFP	19.27 ± 2.57	2078 ± 68.0	107.8	130.5	100
1A9*2-CFP	15.58 ± 1.82	263.7 ± 8.304^###^	16.92^###^	20.48	15.70
1A9*3-CFP	48.11 ± 6.05	600.7 ± 29.6^###^	12.49^###^	15.12	11.59
1A9*5-CFP	22.41 ± 1.99	408.7 ± 11.0^###^	18.24^###^	22.08	16.92

^*^represents compared with UGT1A9*1-HA, while ^#^represents compared with UGT1A9*1-CFP.Data are the mean ± SD of three independent determinations, and the asterisks indicate differences that are statistically significant (^***^P < 0.0001, ^###^*P* < 0.0001).

**Table 3:** Kinetic parameters for quercetin-7-glucuronide by UGT1A9 double expression systems (n = 3).

1A9*N-1A9*N	*K*_m_ (μmol/L)	*K*_si_ (μmol/L)	*V*_max_ (pmol/min/unit)	*CL*_int_ (μL/min/unit)	% of 1A9*1-HA	% of 1A9*1-CFP
1A9*1-CFP + 1A9*2-HA	14.43 ± 1.09		519.0 ± 9.7	35.97^**^	43.55	33.37
1A9*1-CFP + 1A9*3-HA	39.86 ± 9.74	254.0 ± 107.9	2977 ± 448.6	74.69^*^	90.42	69.29
1A9*1-CFP + 1A9*5-HA	31.97 ± 8.93	210.0 ± 91.6	1040 ± 173.0	32.53	39.38	30.18
1A9*2-CFP + 1A9*3-HA	34.33 ± 5.82		675.0 ± 39.8	19.66^**^	23.80	18.24
1A9*2-CFP + 1A9*5-HA	12.92 ± 1.24		145.7 ± 3.6	11.27^***^	13.64	10.45
1A9*3-CFP + 1A9*5-HA	34.66 ± 2.73		514.2 ± 14.2	14.8^**^	17.92	13.73

^*^reprensts the *CL*_int_ of double expression compared with that of the single expression with the two single expression plus together in the bracket. Data are the mean ± SD of three independent determinations, and the asterisks indicate differences that are statistically significant (^***^P < 0.001, ^**^P < 0.005, ^*^P < 0.05).
