# Supplementary material for: Dimerization of human uridine diphosphate glucuronosyltransferase allozymes 1A1 and 1A9 alters their quercetin glucuronidation activities
Source: Sci Rep. 2016 Mar 30;6:23763. doi: 10.1038/srep23763 (PMC4837415; doi:10.1038/srep23763)
Supplement: Supplementary Information [file srep23763-s1.pdf]

Supporting Information

Dimerization of human uridine diphosphate glucuronosyltransferase allozymes 1A1 and 1A9 alters their quercetin glucuronidation activities

Yan-Qing Liu†, Ling-Min Yuan†, Zhang-Zhao Gao, Yong-Sheng Xiao, Hong-Ying Sun, Lu-Shan Yu *& Su Zeng*

Institute of Drug Metabolism and Pharmaceutical Analysis, Zhejiang Province Key Laboratory of Anti-Cancer Drug Research, College of Pharmaceutical Sciences, Zhejiang University, Hangzhou 310058, China

† These authors contributed equally to the work reported in this manuscript.

* Corresponding author: Lushan Yu, Associate Professor. Address: College of Pharmaceutical Sciences, Zhejiang University, Hangzhou, China. Tel: +86 571 88208407; fax: +86 571 88208407; E-mail: yuls@zju.edu.cn; Su Zeng, Professor. Address: College of Pharmaceutical Sciences, Zhejiang University, Hangzhou, China. Tel: +86 571 88208405; fax: +86 571 88208405; E-mail: zengsu@zju.edu.cn

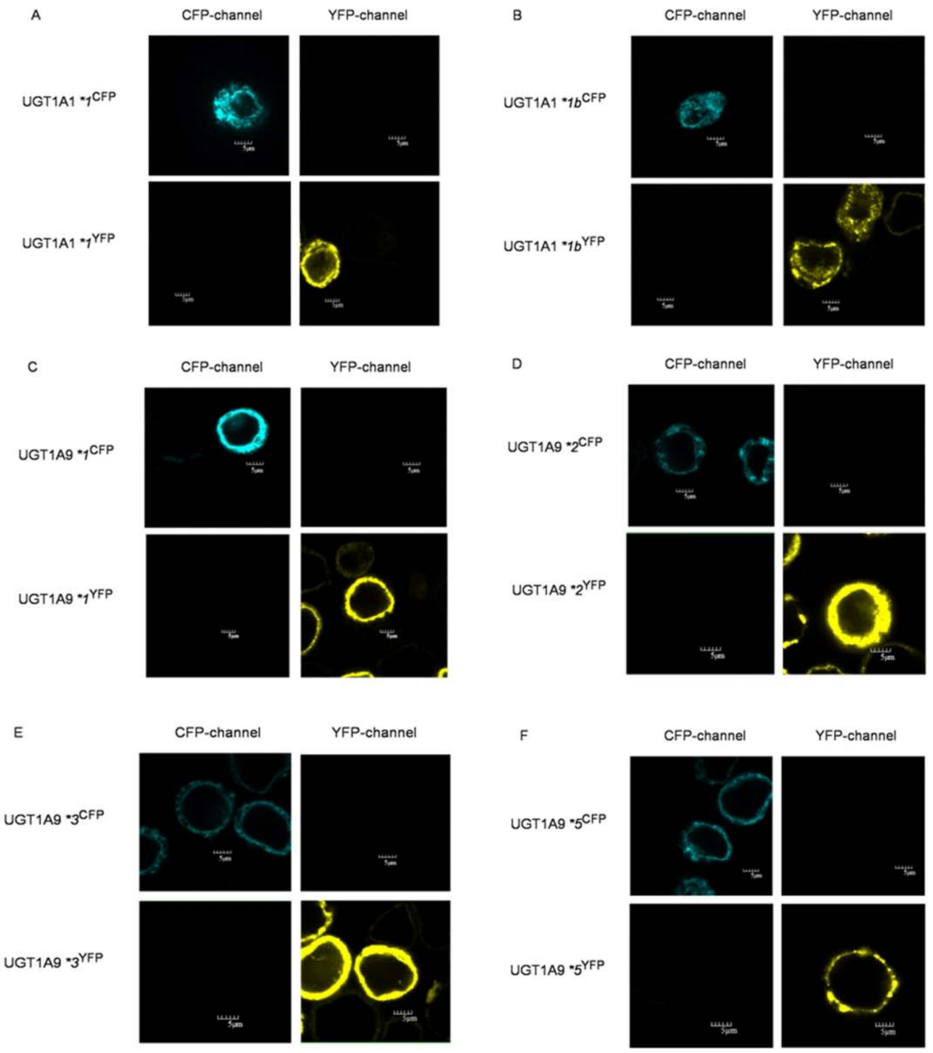

Figure S1. Fluorescence by each UGT1A-CFP and UGT1A-YFP fusion protein in Sf9 cells. Sf9 cells infected with recombinant UGT1A1*1-CFP/YFP *Baculovirus* (A), UGT1A1*1b-CFP/YFP *Baculovirus* (B), UGT1A9*1-CFP/YFP *Baculovirus* (C), UGT1A9*2-CFP/YFP *Baculovirus* (D), UGT1A9*3-CFP/YFP *Baculovirus* (E) and UGT1A9*5-CFP/YFP *Baculovirus* (F). The CFP-tagged proteins were only clearly visible in the CFP channel with no bleed-through in the YFP channel. Conversely, YFP-tagged proteins were only clearly visible in the YFP channel.

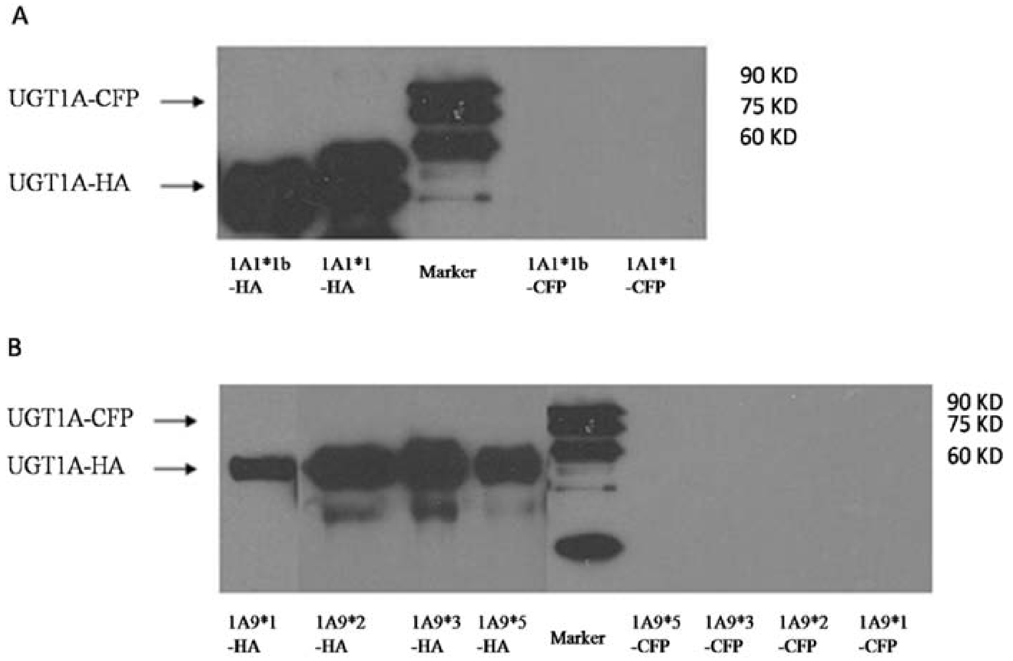

Figure S2. The anti-HA beads specifically immunoprecipitated with the HA-tagged allozyme proteins. To monitor the specificity of the proteins immunoprecipitated with the anti-HA beads, Sf9 cells were transfected with either UGT1A1*N-HA(56 kDa)/CFP (81 kDa) (A) recombinant virus or UGT1A9*N-HA (56 kDa) /CFP (81 kDa) (B) recombinant virus.

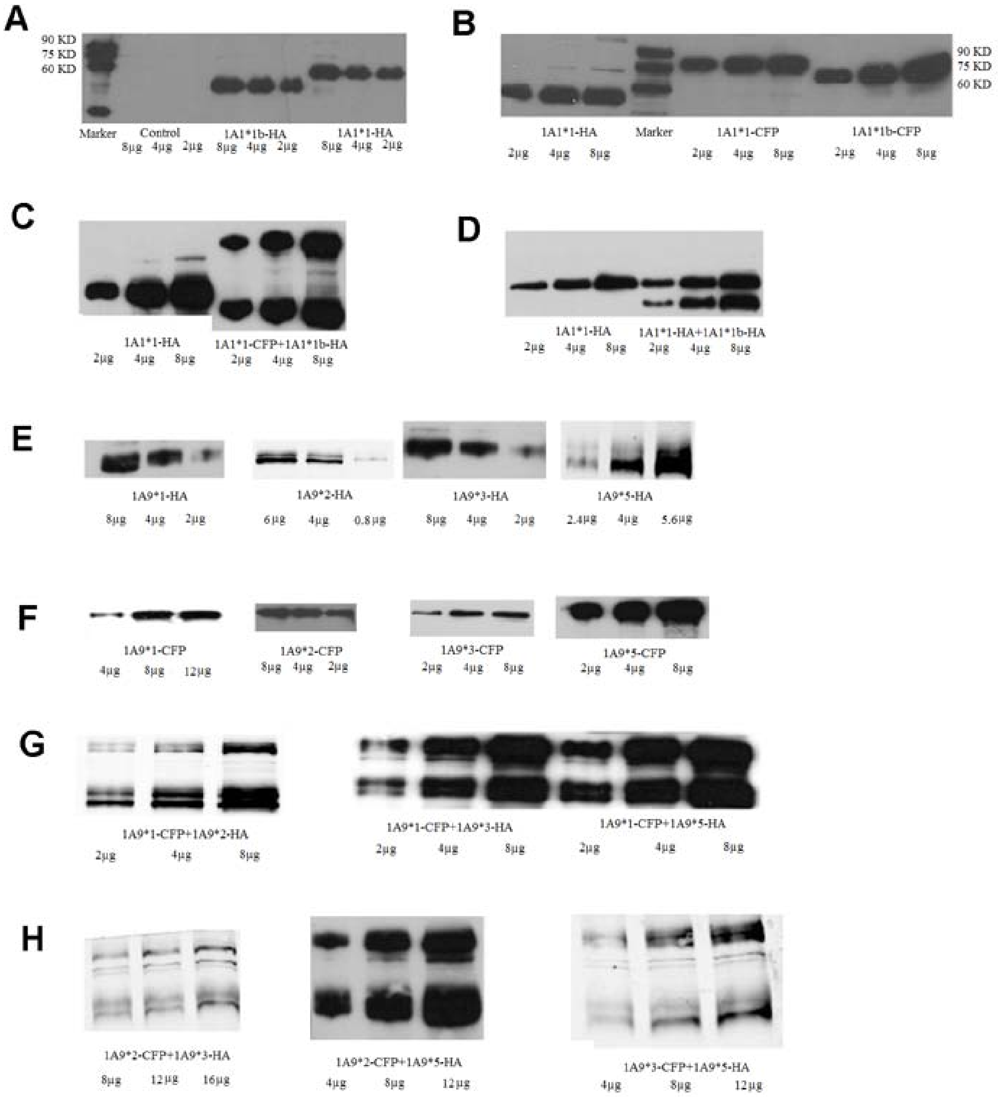

Figure S3. The expression levels of UGT1A allozymes in the single- and double-expression systems. The expression levels of UGT1A1*N (A‒D) and UGT1A9*N (F−I) proteins were determined by immunoblot analysis. The total cell homogenates from Bac-to-Bac expression systems (2 to 8 μg for UGT1A1 and 0.8 to 16 μg for UGT1A9) were subjected to 10% SDS-PAGE and the membranes were probed with anti-UGT1A antibody. The relative expressions were measured using Quantity One 1-D analysis software (E and J). Columns are the mean ± standard deviation (SD) from three independent determinations.

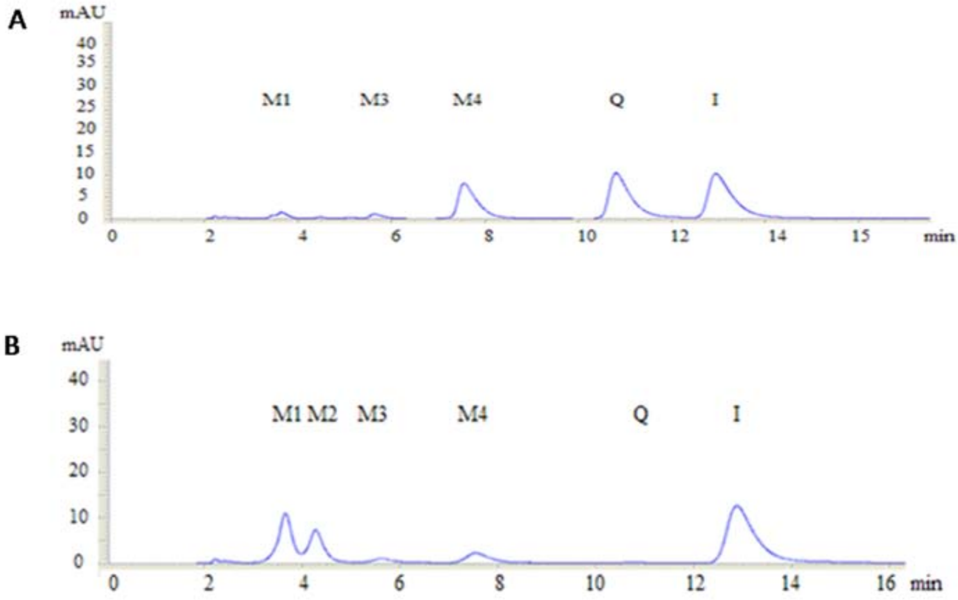

Figure S4. Representative high-pressure liquid chromatography (HPLC) chromatograms of quercetin incubated with UGT1A1*1-HA and UGT1A9*1-HA. The reaction mixture consisted of a total volume of 100 µL containing Tris-HCl (pH = 7.4, 0.1 M), $MgCl_2$ (10 mM), UDPGA (5 mM), alamethicin (50 µg/mg of protein), total cell homogenates expressing UGT1A1*1-HA or UGT1A9*1-HA (1 mg/mL), and quercetin (0.1 mM). The incubation time was 30 min. Quercetin was mainly converted into M1, M3, and M4 by UGT1A1 (A) and M1, M2, and M4 by UGT1A9 (B). “Q” represents quercetin and “I” represents luteolin (internal standard).

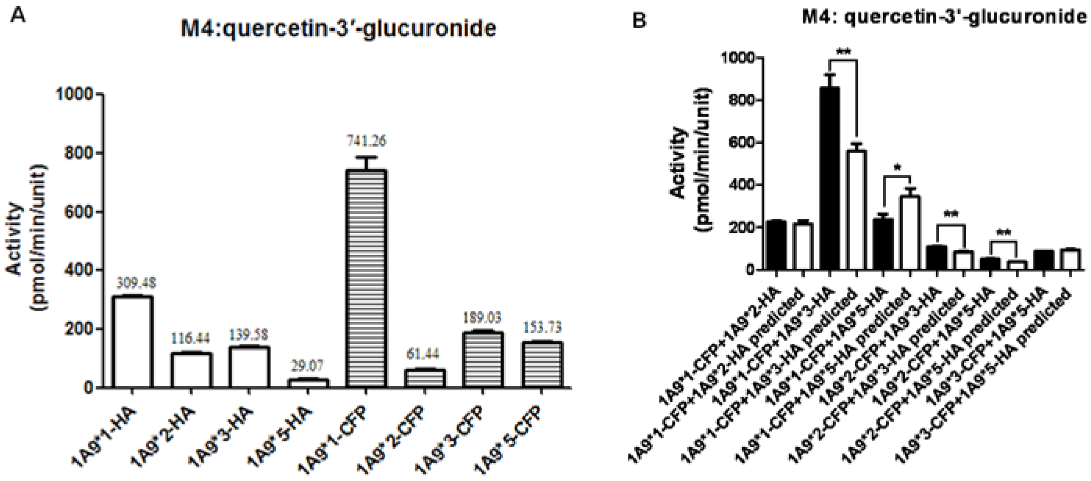

Figure S5. Enzyme activity in quercetin-3′-glucuronidation by UGT1A9*N-HA, UGT1A9*N-CFP, and UGT1A9*N-CFP+UGT1A9*N-HA. Data are presented as mean ± SD of three independent determinations, and the asterisks indicate differences that are statistically significant (****P* < 0.0001, ***P* < 0.005, **P* < 0.05).

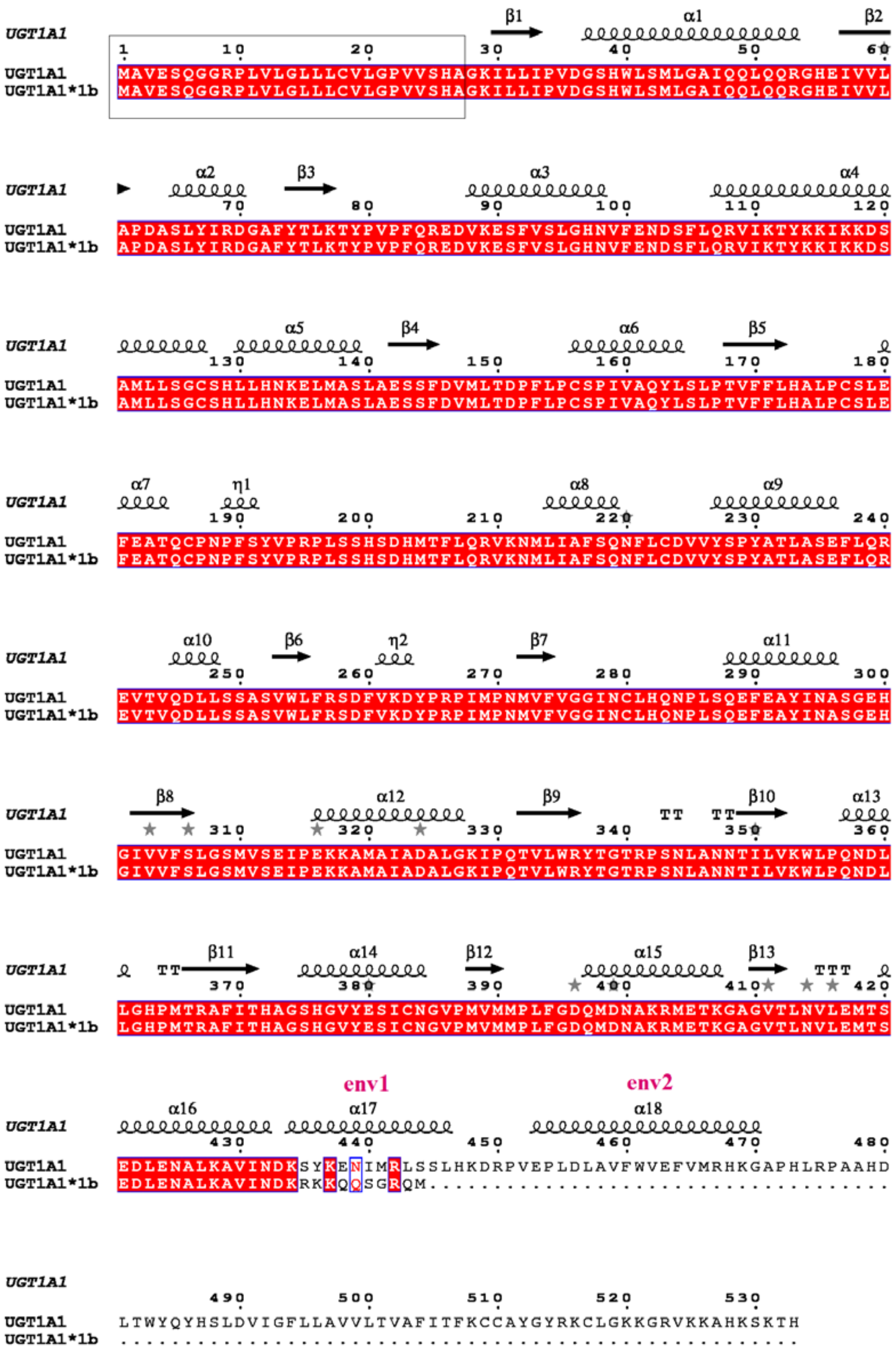

Figure S6. Structure-based sequence alignment of UGT1A1*1 and UGT1A1*1b. The secondary structure elements observed in the UGT1A1 model structure are shown above the alignment [1]. The signal peptide is boxed. This figure was produced with END script [2].

Table S1. Kinetic parameters for quercetin-3-glucuronide by UGT1A9 single expression systems.

1A9*N-1A9*N	K_m (μmol/L)	K_{si} (μmol/L)	V_{max} (pmol/min/unit)	CL_{int} (μL/min/unit)	% of 1A9*1-HA	% of 1A9*1-CFP
1A9*1-HA	6.93 ± 1.52		600.4 ± 27.61	86.64	100	37.87
1A9*2-HA	3.64 ± 0.41		$211.0 \pm 3.5^{***}$	57.89^{*}	66.82	25.30
1A9*3-HA	21.85 ± 4.80	195.7 ± 61.8	$467.3 \pm 57.3^{*}$	21.39^{**}	24.69	9.35
1A9*5-HA	7.21 ± 1.39		$34.75 \pm 1.43^{***}$	4.82^{***}	5.56	2.11
1A9*1-CFP	7.39 ± 0.97		1691 ± 44.0	228.8	264.1	100
1A9*2-CFP	21.02 ± 6.99	233.0 ± 120.4	$175.5 \pm 31.8^{\#\#\#}$	$8.35^{\#\#\#}$	9.64	3.65
1A9*3-CFP	34.39 ± 7.55	139.5 ± 40.2	$688.3 \pm 96.1^{\#\#\#}$	$20.01^{\#\#\#}$	23.10	8.75
1A9*5-CFP	34.65 ± 9.19	211.5 ± 87.7	$448.7 \pm 73.8^{\#\#\#}$	$12.95^{\#\#\#}$	14.95	5.66

* represents compared with UGT1A9*1-HA, while $^{\#}$ represents compared with UGT1A9*1-CFP.Data are the mean ± SD of three independent determinations, and the asterisks indicate differences that are statistically significant ($^{***}P<0.0001$, $^{**}P<0.005$, $^{*}P<0.05$; $^{\#\#\#}P<0.0001$, $^{\#}P<0.05$).

Table S2. Kinetic parameters for quercetin-3-glucuronide by UGT1A9 double expression systems.

1A9*N-1A9*N	K_m (μmol/L)	K_{si} (μmol/L)	V_{max} (pmol/min/unit)	CL_{int} (μL/min/unit)	% of 1A9*1-HA	% of 1A9*1-CFP
1A9*1-CFP+1A9*2-HA	2.09 ± 0.28		743.7 ± 10.9	356.0***	410.9	155.6
1A9*1-CFP+1A9*3-HA	5.28 ± 0.96		1816 ± 57.9	343.9***	396.9	150.3
1A9*1-CFP+1A9*5-HA	12.94 ± 2.52	232.9 ± 59.8	788.0 ± 65.5	60.90*	70.29	26.62
1A9*2-CFP+1A9*3-HA	11.52 ± 1.72	278.8 ± 65.3	475.4 ± 32.3	41.27***	47.63	18.04
1A9*2-CFP+1A9*5-HA	13.83 ± 2.22	263.0 ± 66.0	227.4 ± 17.6	16.44**	18.98	7.19
1A9*3-CFP+1A9*5-HA	8.23 ± 1.25		278.3 ± 9.4	33.8*	39.01	14.77

* reprensts the CL_{int} of double expression compared with that of the single expression with the two single expression plus together in the bracket. Data are the mean ± SD of three independent determinations, and the asterisks indicate differences that are statistically significant ($^{***}P<0.0001$, $^{**}P<0.005$, $^{*}P<0.05$).

Table S3. Plasmid constructs used for FRET analysis and co-immunoprecipitation test.

	pFastBac1-UGT1A-CFP plasmids	pFastBac1- UGT1A -YFP plasmids	pFastBac1- UGT1A -HA plasmids
1	*UGT1A 1-CFP*	*UGT1A 1-YFP*	*UGT1A 1-HA*
2	*UGT1A 1*1b-CFP*	*UGT1A 1*1b -YFP*	*UGT1A 1*1b -HA*
3	*UGT1A 9*1-CFP*	*UGT1A 9*1-YFP*	*UGT1A 9*1-HA*
4	*UGT1A 9*2-CFP*	*UGT1A 9*2-YFP*	*UGT1A 9*2-HA*
5	*UGT1A 9*3-CFP*	*UGT1A 9*3-YFP*	*UGT1A 9*3-HA*
6	*UGT1A 9*5-CFP*	*UGT1A 9*5-YFP*	*UGT1A 9*5-HA*

References

1. Laakkonen, L. & Finel, M. A molecular model of the human UDP-glucuronosyltransferase 1A1, its membrane orientation, and the interactions between different parts of the enzyme. *Mol. Pharmacol*. **77**, 931-939 (2010).
2. Robert, X. & Gouet, P. Deciphering key features in protein structures with the new ENDscript server. *Nucleic Acids Res*. **42**(Web Server issue), W320-324(2014).
